# Effect of an emergency/planned cesarean section on the perinatal outcomes of pregnant women with placenta accreta spectrum and their neonates: a retrospective study cohort

**DOI:** 10.3389/fsurg.2025.1603425

**Published:** 2025-08-20

**Authors:** Duan Duan, Sumei Wei, Dongmei Tang, Shimao Zhang, Jinzhu Fu, Linbo Cheng, Mi Su, Wenyi Lin, Wen Xiong

**Affiliations:** Chengdu Women’s and Children’s Central Hospital, School of Medicine, University of Electronic Science and Technology of China, Chengdu, China

**Keywords:** placenta accreta spectrum disorders, emergency cesarean section, planned cesarean section, perinatal outcomes, perinatal fetal outcomes, intraoperative blood loss, multidisciplinary treatment

## Abstract

**Background:**

Placenta accreta spectrum (PAS) is an obstetric condition. This study analyzes the outcomes of PAS parturients and their newborns undergoing emergency cesarean sections as opposed to planned cesarean sections.

**Methods:**

In this research, we conduct a thorough retrospective analysis of 345 patients with placenta accreta at a single medical center. The patients were systematically categorized into two groups based on the type of surgical procedure: emergency cesarean sections and planned cesarean sections. A univariate analysis was performed to compare the outcomes associated with each approach. Furthermore, a logistic regression analysis was used to identify independent risk factors related to emergency surgeries. To further enhance our understanding, a multiple linear regression analysis was employed to determine the key factors influencing intraoperative bleeding. The selection of covariates for the regression analyses was carefully guided by previously reported influencing factors, as well as the significant differences highlighted in the univariate analysis. Missing data were addressed using multiple imputation based on chained equations to reduce potential bias and preserve analytical integrity. The study enrolled all women with PAS between August 2019 and February 2022. Ethical approval for this study was obtained from the Ethics Committee of Chengdu Women's and Children's Center Hospital (Ethics DOI: 201830). All participants provided written informed consent.

**Results:**

The amount of bleeding and allogeneic blood transfusion in mothers in the emergency group was higher than that in the planned group. In the newborns in the emergency group, birth weight, length, and gestational age were lower than those in the planned group. The analysis found that the operation mode (*B* = 158.174, *P* = 0.043, 95% CI: 4.99–311.358) and the duration of operation (min) (*B* = 13.348, *P* < 0.001, 95% CI: 11.878–14.817) significantly affected the amount of intraoperative bleeding.

**Conclusions:**

The perinatal outcomes and perinatal infant outcomes in the emergency group were worse than those in the planned group, as exemplified by a lower neonatal birth weight, shorter body length, smaller gestational age at birth, and higher incidence of severe neonatal asphyxia. An emergency cesarean section may increase the amount of bleeding during a cesarean section in patients with PAS. A multidisciplinary team managing such patients can achieve favorable outcomes.

**Clinical Trial registration:**

identifier (ChiCTR2100054068)

## 1 Background

Placenta accreta spectrum (PAS) disorders are diseases in which placental villi invade the myometrium of the uterus to varying degrees (1). PAS can be divided into three types: adherent placenta accreta, placenta increta, and placenta percreta, according to the depth of the placental villi invading the myometrium (2–5).

The rate of prevalence of PAS is 0.01%–1.1% (6). A meta-analysis has revealed that risk factors associated with PAS include obesity, maternal age >over 35 years, prepregnancy or prenatal smoking, placenta previa, a history of prior cesarean delivery, placenta previa combined with a history of cesarean section, a history of uterine surgery, abortion and curettage, *in vitro* fertilization (IVF) pregnancy, and a short interpregnancy interval following cesarean section (7). PAS is a common cause of severe postpartum hemorrhage, hysterectomy, and serious maternal complications, leading to increased mortality (8–10).

Ultrasound and nuclear magnetic resonance are both sensitive and specific enough to diagnose pulmonary arterial hypertension (PAH). The presence of three or more imaging signs can significantly enhance the accuracy of the diagnosis. The following US signs were evaluated: intraplacental lacunae, loss of the retroplacental hypoechoic zone, myometrial thinning <1 mm, bladder wall interruption, placental bulging, bridging vessels, and the hypervascularity of the uterovesical or retroplacental space. The MRI signs that were evaluated were intraplacental dark T2 bands, placental bulging, loss of the retroplacental hypointense line on T2 images, myometrial thinning, bladder wall interruption, focal exophytic placental mass, and abnormal vascularization of the placental bed (11). Emergency surgery often involves higher blood loss and blood transfusion rates than planned surgery, and the gestational age of perinatal infants is lower (12). Cesarean section is the preferred mode of delivery for such patients, but how to reduce the amount of intraoperative blood loss and improve maternal perinatal and perinatal infant outcomes is an urgent problem in obstetrics. Patients with PAS should connect with a multidisciplinary team (MDT) before delivery to assess high-risk factors. Hospitalization is essential to determine the best timing for a cesarean section. This standardized procedure can reduce risks of bleeding, premature delivery, and fetal asphyxia, while allowing adequate time for preoperative preparation and minimizing severe maternal complications (8, 10).

We have established a dedicated MDT in our obstetrics department to address the problem of PAS and ensure the safety of mothers and their babies. When prenatal concerns about PAS arise, our MDT creates personalized treatment plans and selects the best timing for delivery. Recognizing the risk of bleeding and the potential need for transfusions, we have adopted a patient blood management (PBM) approach. This innovative strategy aims to minimize red blood cell (RBC) loss during critical phases, enhancing patient safety and outcomes (13). Among patients with PAS, whether cesarean delivery is planned or urgent, their perinatal outcomes and perinatal infant outcomes are still inconclusive. In addition, there is little discussion about how to reduce bleeding in patients with PAS and improve the prognosis of the mother and the baby.

Between August 2019 and February 2022, 345 patients with PAS at Chengdu Women's and Children's Central Hospital, all diagnosed with cesarean sections, were analyzed and divided into two groups: emergency operations and planned operations. This study aimed to evaluate the perinatal outcomes and perinatal infant outcomes of PAS parturients under emergency and planned cesarean sections, investigate the factors influencing intraoperative bleeding, and explore the effect of programmed management of placenta accreta disease on PAS.

## 2 Methods

### 2.1 Comparison of baseline data between groups

Research object and grouping: Between August 2019 and February 2022, 345 patients with PAS diagnosed and undergoing cesarean section were enrolled. According to whether the operation was an emergency cesarean section or a planned cesarean section, 39 patients were included in the emergency group and 306 patients in the planned group. The perioperative data of the patients were collected.

The baseline data of this experiment included age, PAS type, number of pregnancies (G), number of deliveries (P), number of previous cesarean sections, and number of induced abortions. Second, there were many high-risk pregnancy factors, including intrahepatic cholestasis of pregnancy (ICP), gestational diabetes mellitus (GDM), hypertensive disorder complicating pregnancy (HDCP), scarred uterus, multiple pregnancy, polyhydramnios, oligohydramnios, marginal umbilical cord attachment, and premature rupture of membranes (PROM). In addition, the data included preoperative blood loss and hemoglobin (HB).

### 2.2 Comparison of perioperative data between the two surgical approaches

We included the following data during the cesarean section: temporary occlusion of the abdominal aorta with a balloon catheter before the operation, the duration of the operation (min), bladder injury, hysterectomy, blood loss (ml) during the operation, uterine artery embolism after the operation, and whether a hole was made through the placenta to let the placenta enter. Finally, there are ways to address bleeding: intrauterine balloon and bundling of the lower uterine segment. The patient was forced to receive the total amount of blood products: red blood cell suspension (U), fresh frozen plasma (ml), and autologous blood transfusion (ml). The birth situation of newborns was also described, including birth weight (g), body length (cm), neonatal asphyxia, and gestational age. We also considered the unplanned second operation, 24 h blood loss (ml), and HB (g/L) 3 days after the operation.

### 2.3 Statistical methods

SPSS 26.0 statistical software was used to analyze the data. After testing the measurement data to conform to normal distribution and the homogeneity of variance, we expressed the data as means ± SD. An independent sample t-test was used to compare the two groups, and the *P*-value was assumed to be the equal variance result. If the measurement data conformed to normal distribution but the variance was uneven, the *p*-value selection did not assume an equal variance result. We expressed the non-normal distribution of measurement data by median and quartile (Q25, Q75) and used the Mann‒Whitney *U* test to compare the groups. Data were expressed as frequencies (percentages), and the chi-square test was used for making comparisons between the groups. After a preoperative single-factor comparison, the factor (*P* < 0.1) was included in the logistic regression analysis model. We established a multivariate linear regression model that took intraoperative blood loss as the dependent variable and several factors as independent variables. These independent variables are summarized according to our clinical experience, and they may significantly affect the amount of intraoperative bleeding. A value of *p* < 0.05 was used to indicate statistically significant differences.

## 3 Results

### 3.1 Comparison of baseline data between the two surgical procedures

There were 31 (28,34) patients in the emergency group and 32 (29,35) patients in the planned operation group. The age difference between the two groups was statistically significant. In the emergency group, there were 27 patients with placental adhesion (69.2%), nine with placental implantation (23.1%), and three with placental penetration (7.7%). There were 194 patients with planned placental adhesion (63.4%), 102 patients with placenta accreta (33.3%), and 10 with placental penetration (3.3%). We found that other data in the emergency group were significantly different from those in the planned group, including the number of pregnancies (≤1 time 23.1% vs. 11.1%; >1 time 76.9% vs. 88.9%; *p* = 0.0410), induced abortion times (≤1 time 69.2% vs. 52.3%; >1 time 3.9% vs. 47.7%; *p* = 0.04), scarred uterus (20.5% vs. 51%; *p* < 0.001), preoperative bleeding time (≤2 times, 61.5% vs. 87.3%; >2 times, 38.5% vs. 12.7%; *p* < 0.001), and preoperative HB (g/L) (106.1 ± 13.782 vs. 114.13 ± 11.681, *p* < 0.001) (Table 1).

**Table 1:** Preoperative data.

Characteristics	Emergency (*n* = 39)	Planned (*n* = 306)	*P*
Age (years)	31 (28,34)	32 (29,35)	0.043^a^
PAS type			0.162^b^
Adherent placenta accreta	27 (69.2)	194 (63.4)	
Placenta increta	9 (23.1)	102 (33.3)	
Placenta percreta	3 (7.7)	10 (3.3)	
G (*n*)			0.041^b^
≤1	9 (23.1)	34 (11.1)	
>1	30 (76.9）	272 (88.9)	
P (*n*)			0.751^b^
≤1	20 (51.3)	149 (48.7)	
>1	19 (48.7)	157 (51.3)	
Previous cesarean section times (*n*)			0.753^b^
≤1	36 (92.3)	284 (92.8)	
>1	3 (7.7)	22 (7.2)	
Induced abortions (*n*)			0.045^b^
≤1	27 (69.2)	160 (52.3)	
>1	12 (3.9)	146 (47.7)	
ICP	2 (5.1)	14 (4.6)	0.7^b^
GDM	9 (23.1)	55 (18)	0.44^b^
HDCP	0	17 (5.6)	0.236^b^
Scarred uterus	8 (20.5)	156 (51)	<0.001^b^
Multiple pregnancy	2 (5.1)	7 (2.3)	0.27^b^
Polyhydramnios	2 (5.1)	2 (0.7)	0.065^b^
Oligoamnios	0	7 (2.3)	1^b^
Marginal umbilical cord attachment	4 (10.3)	12 (3.9)	0.093^b^
PROM	3 (7.7)	19 (6.2)	0.726^b^
Preoperative bleeding times (*n*)			<0.001^b^
≤2	24 (61.5)	267 (87.3)	
>2	15 (38.5)	39 (12.7)	
HB (g/L) before operation	106.1 ± 13.782	114.13 ± 11.681	<0.001^c^

^a^ Median (lower quartile, upper quartile). Mann‒Whitney *U* test.

^b^ Number (percentage). Chi-squared test.

^c^ Average and standard deviation. Student's *t*-test.

G, number of pregnancies; P, number of deliveries; ICP, intrahepatic cholestasis of pregnancy; GDM, gestational diabetes mellitus; HDCP, hypertensive disorder complicating pregnancy; PROM, premature rupture of membranes; *n*, number; y, year.

### 3.2 Logistics regression analysis of preoperative factors leading to emergency surgery risk factors

In the analysis of preoperative data, we incorporated the single factor with *P* < 0.1 (these factors include age, preoperative bleeding times, pregnancy times, abortion times, scarred uterus, polyhydramnios, marginal umbilical cord attachment, and preoperative hemoglobin amount) into the binary logistics regression model. The results showed that the number of bleeds before the operation (OR: 3.224, CI 95%: 1.38–7.532, *P* < 0.001), scarred uterus (OR: 0.237, CI 95%: 0.094–0.6, *p* = 0.002), polyhydramnios (OR: 11.642, CI 95%: 1.245–108.893, *P* = 0.031), and HB before the operation (OR: 0.94, CI 95%: 0.909–0.972, *p* < 0.001) were independent risk factors for emergency operation (Table 2). The MDT responsible for the care of patients with PAS should prioritize these independent risk factors, as doing so can significantly enhance patient outcomes.

**Table 2:** Logistics regression analysis of preoperative factors leading to emergency surgery risk factors.

Variables		*B*	*P*	OR	CI 95%
Age		−0.021	0.642	0.979	0.897–1.07
Number of bleeds before operation	>2	1.171	0.007	3.224	1.38–7.532
	≤2	0		1	
G	>1	0.152	0.781	1.164	0.399–3.399
	≤1	0		1	
Number of induced abortions	>1	−0.587	0.188	0.556	0.232–1.332
	≤1	0		0	
Scarred uterus	y	−1.438	0.002	0.237	0.094–0.6
	n	0		1	
Polyhydramnios	y	2.455	0.031	11.642	1.245–108.893
	n	0		1	
Marginal umbilical cord attachment	y	1.202	0.087	3.325	0.84–13.155
	n	0		1	
HB (g/L) before operation		−0.062	<0.001	0.94	0.909–0.972

CI, confidence interval; OR, odds ratio; y, yes; n, no.

Single factors (*P* < 0.1) in the univariate analysis of Table 1 were taken as covariates, and the surgical form was taken as a dependent variable to establish a binary logistic regression.

### 3.3 Logistics regression analysis of preoperative factors leading to emergency surgery risk factors

Through intraoperative and postoperative data analyses, we found that the abdominal aorta balloon catheter plus temporary occlusion (10.3% vs. 35.4%, *p* = 0.002) was significantly lower in the emergency group than in the planned group. This situation may arise due to insufficient time for abdominal aortic balloon occlusion during clinically urgent surgeries. Under the condition of no difference in autologous blood transfusion, the total amount of red blood cell suspension [0 (0,4) vs. 0 (0,0), *p* = 0] and plasma [0 (0,0) vs. 0 (0,0), *p* = 0.031] input was significantly different. This finding, when combined with clinical data, better illustrates that excessive blood loss or low hemoglobin levels prior to an emergency cesarean section make it more difficult for mothers to tolerate substantial blood loss within a short period during the procedure. With regard to newborns, the birth weight (G)[2,080 (1,730,2,400) vs. 2,825 (2,500, 3,140), *p* < 0.001] and body length (cm) [45 (42, 48) vs. 49 (47, 50), *p* < 0.001] of the newborn and the gestational age (weeks) [33.29 (31, 35) vs. 36.43 (36,37), *p* < 0.001] were lower in the emergency group than in the planned group. It is reasonable to assume that neonatal birth weight and length may be lower than in the planned group, given the differences in gestational ages, which are lower in the emergency group. Moreover, our study found two patients (5.1%) with severe neonatal asphyxia in the emergency group and 0 patients in the planned group (*P* = 0.001), and the risk of severe neonatal asphyxia in the emergency group was increased. However, the sample size for emergency cesarean sections was only 39 patients, which may result in insufficient statistical power and make the results susceptible to the influence of outliers. We must acknowledge the limitations regarding whether the two patients with severe neonatal asphyxia can truly represent population risks. Future studies should expand the sample size of emergency cesarean sections through multicenter collaboration to validate the findings. HB (g/L) (86.49 ± 10.323 vs. 97.79 ± 15.309; *p* < 0.001) was lower in the emergency group 3 days after the operation. A postoperative pathological examination showed that the incidence of chorioamnionitis in the emergency group was higher than that in the planned group (23.1% vs. 7.8%, *p* = 0.006), as shown in Table 3.

**Table 3:** Comparison of intraoperative conditions between the emergency group and the planned group.

Variables	Emergency (*n* = 39)	Planned (*n* = 306)	*P*
Temporary occlusion of balloon catheter insertion in abdominal aorta	4 (10.3)	108 (35.4)	0.002^a^
Operation duration (min)	50 (44,58)	53 (42,53)	0.525^b^
Uterine artery embolization after operation	2 (5.1)	6 (2)	0.227^a^
Bladder injury	0	3 (1)	1^a^
Hysterectomy	3 (7.7)	18 (5.9)	0.718^a^
Intraoperative blood loss (ml)	800 (600, 1,000)	800 (600, 1,000)	0.756^b^
Punch a hole into the placenta	14 (35.9)	98 (32)	0.717^a^
Balloon packing of uterine cavity	27 (69.2)	218 (71.2)	0.794^a^
Bundle the lower uterine segment	14 (35.9)	105 (34.3)	0.845^a^
Red blood cell suspension (U)	0 (0,4)	0 (0,0)	<0.001^b^
Blood plasma (U)	0 (0,0)	0 (0,0)	0.031^b^
Autologous blood transfusion (U)	0 (0,0)	0 (0,0)	0.834^b^
Birth weight (g)	2,080 (1,730, 2,400)	2,825 (2,500, 3,140)	<0.001^b^
Body length (cm)	45 (42,48)	49 (47,50)	<0.001^b^
Asphyxia neonatorum			0.001^a^
Mild	10 (25.6)	38 (12.4)	
Serious	2 (5.1)	0	
Gestational age of the newborn (week)	33.29 (31,35)	36.43 (36,37)	<0.001^b^
Unscheduled second operation	1 (2.6)	2 (0.7）	0.303^a^
24-h bleeding (ml)	930 (645, 1,330)	845 (700, 1,162.5)	0.705^b^
HB (g/L) 3 days after operation	86.49 ± 10.323	97.79 ± 15.309	<0.001^c^
Chorioamnionitis	9(23.1)	24(7.8)	0.006^a^

^a^ Number (percentage). Chi-squared test.

^b^ Median (lower quartile, upper quartile).Mann‒Whitney *U* test.

^c^ Average and standard deviation. Student's *t*-test.

### 3.4 Multiple linear regression model with intraoperative blood loss as the dependent variable

We established a multiple linear regression model with intraoperative blood loss as the dependent variable and several factors as independent variables. These independent variables are summarized according to our clinical experience, and they may significantly affect the amount of intraoperative bleeding. These independent variables include the following: operation form, P, number of cesarean sections, number of induced abortions, ICP, HDCP, GDM, multiple pregnancies, polyhydramnios, operation duration, temporary occlusion of balloon catheter insertion in the abdominal aorta, bladder injury, PAS type, a hole punched into the placenta, and chorioamnionitis (Table 4).

1. The type of operation was an independent factor of intraoperative blood loss (*B* = 158.174, *P* = 0.043, 95% CI: 4.99–311.358). Compared with the planned operation, the emergency operation increased intraoperative blood loss by 158.174 ml.
2. The duration of operation (min) was an independent influencing factor of intraoperative blood loss (*B* = 13.348, *P* < 0.001, 95% CI: 11.878–14.817). This showed that every minute of increased operation time was associated with an increase in intraoperative bleeding of approximately 13 ml.
3. PAS classification was an independent influencing factor of intraoperative blood loss (*B* = 242.515, *p* < 0.001, 95% CI: 142.32–342.71). A higher PAS classification was associated with more intraoperative blood loss.

**Table 4:** Multiple linear regression model with intraoperative blood loss as the dependent variable.

Parameter	*B*	*P*	95% CI	VIF
(Constant)	−171.732	0.021	−316.899 to −26.566	
Operation form (0 = planned; 1 = emergency)	158.174	0.043	4.99–311.358	1.138
P	−16.83	0.639	−87.423 to 53.764	1.802
Number of cesarean sections	−81.31	0.094	−176.63 to 14.009	2.126
Number of induced abortions	13.099	0.438	−20.092 to 46.289	1.109
ICP	55.959	0.636	−176.611 to 288.53	1.157
HDCP	−56.357	0.607	−271.795 to 159.082	1.052
GDM	−102.089	0.101	−224.141 to 19.963	1.089
Multiple pregnancy (0 = n; 1 = y)	146.759	0.339	−154.661 to 448.179	1.116
Polyhydramnios (0 = n; 1 = y)	120.967	0.591	−321.042 to 562.976	1.083
Operation duration (min)	13.348	<0.001	11.878–14.817	1.658
Temporary occlusion of balloon catheter insertion in abdominal aorta (0 = n; 1 = y)	−66.644	0.255	−181.737 to 48.45	1.405
Bladder injury (0 = n; 1 = y)	−398.187	0.379	−1,288.135 to 491.762	1.107
PAS type (1 = adhesion; 2 = Implantation; 3 = penetration)	242.515	<0.001	142.32–342.71	1.528
Punch a hole into the placenta (0 = none; 1 = yes)	41.912	0.417	−59.472 to 143.297	1.09
Chorioamnionitis (0 = n; 1 = y)	−105.29	0.191	−263.232 to 52.652	1.044
*F*		42.866		
*P*		<0.001		
*R* ^2^		0.662		
Dependent variable: intraoperative blood loss (ml)				

B, unstandardized coefficients; 95% CI, 95.0% confidence interval for *B*; y, yes; n, no; P, number of deliveries; ICP, intrahepatic cholestasis of pregnancy; GDM, gestational diabetes mellitus; HDCP, hypertensive disorder complicating pregnancy.

Based on the above calculation results.

**Figure 1:**
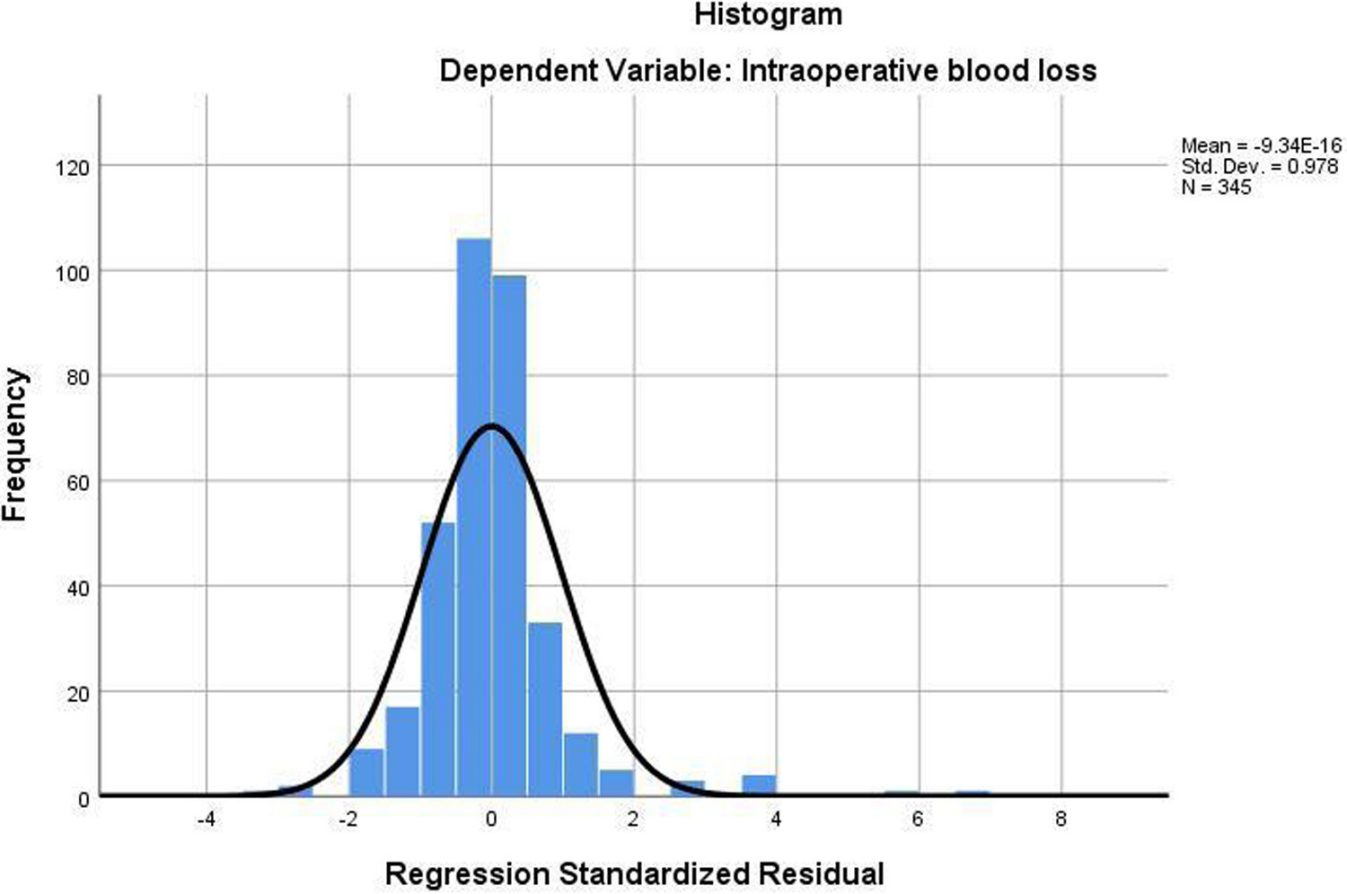
The residuals of the regression equation are as follows.

The above regression analysis results are accurate and reliable—the fitting degree of the regression Equation *R*^2^ = 0.662 was good. Meanwhile, the regression equation was significant (*F* = 42.866, *P* < 0.001), indicating that this equation was meaningful (Figure 1).

## 4 Discussion

Upon analyzing the 345 patients, we differentiated between those undergoing emergency procedures and those with scheduled interventions to examine maternal and neonatal outcomes during the perioperative period. Our findings indicated that the emergency group experienced significantly worse outcomes, primarily due to increased preoperative bleeding and a greater dependence on allogeneic blood products. Infants in the emergency group also exhibited lower birth weights, reduced heights, and shorter gestational ages, coupled with a heightened risk of severe neonatal asphyxia. These results underscore the serious complications linked to emergency cesarean sections, particularly for patients diagnosed with PAS, highlighting the necessity for meticulous management in high-risk scenarios.

Patients with PAS face a heightened risk of significant postpartum hemorrhage, which can occur in various settings and complicate accurate blood loss estimation. Our analysis of preoperative hemoglobin levels suggests that lower hemoglobin levels are associated with increased vaginal blood loss in these patients. According to the results of the preoperative data comparison, the number of bleeding events before the operation (>2 times) was 3.224 times that of the emergency cesarean section before the operation (≤2 times). For patients with placental implantation disorders, understanding the risk factors for postpartum hemorrhage is crucial. By enhancing prenatal care and early intervention, we can reduce the incidence of postpartum hemorrhage and improve maternal outcomes. Early integration of patients with PAS into MDT management enables an early detection of symptoms, streamlines hospitalization, and allows for proactive measures. This approach aims to minimize vaginal bleeding before delivery, ultimately reducing the need for emergency cesarean sections and enhancing the safety of both mothers and their babies.

The findings reported by Shamshirsaz et al. on the comparison between planned and emergency cesarean hysterectomy indicated an increased rate of blood product infusion, a decrease in the Apgar scores of newborns, and an elevated incidence of respiratory distress syndrome (12, 14). These outcomes are consistent with those of our study. In the emergency group, there was an increase in blood transfusion volume, earlier delivery of the newborn, lower birth weight and length, and a higher incidence of severe asphyxia compared with the planned group. We also observed that the emergency group had a higher incidence of chorioamnionitis. Intrauterine infection may lead to fetal hypoxia, induce uterine contractions, and cause preoperative bleeding.

We established a multivariate linear regression model with intraoperative blood loss as the dependent variable. We found that planned emergency operations increased the bleeding volume by 158.174 ml, whereas longer operation times led to increased bleeding during the procedure. A prenatal diagnosis of PAS is associated with a lower frequency of blood transfusions (15). The amount of bleeding in the planned group was less than that in the emergency group, which may be attributed to the MDT management at our center. Patients can participate in MDT management if there is an early prenatal diagnosis of PAS. Throughout the period of pregnancy, patients are put under MDT management, and prenatal care medical staff can dynamically assess the severity of the patients' condition. Prior to surgery, the degree of placenta implantation is evaluated using an ultrasound score, and MRI is used to clarify the depth and location of placenta implantation and its relationship with the bladder. In the absence of risk factors for preterm delivery in women with placenta accreta spectrum, planned delivery at 35+0 to 36+6 weeks of gestation provides the best balance between fetal maturity and the risk of unscheduled delivery (16). We ensure adequate preoperative preparations, including blood preparation, ultrasound-assisted incision localization, antibiotic selection, and the arrangement of the operating team. After surgery, we decide whether to transfer the patient to the general ward based on their condition. ICU doctors and obstetricians jointly manage patients, adopting a dual approach. Some emergency operations can be avoided as much as possible through this management approach. By reducing bleeding and delaying the gestational age, we aim to improve the perinatal outcomes for both the mother and the baby.

MDT members should include doctors from the following departments: obstetrics and gynecology, neonatology, anesthesiology, radiology, interventional departments, and other related departments. Under the premise of full communication between doctors and patients, full planning, prenatal PAS testing, and multidisciplinary teams can reduce the incidence of serious maternal diseases (17, 18).

This study offers several advantages, including a relatively large sample size. It encompassed numerous factors during the perioperative period, and all patients were treated at the same research center. Our hospital has also established an MDT. The retrospective analysis of past cases and the comparison between planned and emergency cesarean sections underscore the crucial role of MDT management. However, this study has limitations due to its modest sample size and the risk of selection bias, as it originates from one hospital specializing in obstetrics and gynecology. In primary hospitals lacking MDT capabilities, implementation is limited. Therefore, a further extensive, multicenter analysis is essential for improving patient outcomes.

## 5 Conclusion

Our research results show that emergency cesarean sections in patients with PAS have worse perinatal and perinatal infant outcomes than planned cesarean sections.

## Data Availability

The original contributions presented in the study are included in the article/Supplementary Material, and further inquiries can be directed to the corresponding author.
